# Service evaluation of R90 bleeding and platelet disorders gene panel in thrombocytopenia cases

**DOI:** 10.1111/bjh.19947

**Published:** 2024-12-09

**Authors:** G. Gurumurthy, L. Reynolds, M. Sutherland, J. Thachil, J. Grainger

**Affiliations:** ^1^ University of Manchester Manchester UK; ^2^ Royal Manchester Children's Hospital Manchester UK; ^3^ North West Genomic Laboratory Hub Manchester University NHS Foundation Trust Manchester UK; ^4^ Manchester University NHS Foundation Trust Manchester UK

**Keywords:** genetics, ITP, R90, thrombocytopenia

## Abstract

This study examines the R90 bleeding and platelet disorders gene panel's utility in thrombocytopenia. The study analysed the correlations between the clinical features of patients with thrombocytopenia and genetic outcomes. The diagnostic yield was 46.6% (41/88) for the overall panel for all patients referred locally. Thrombocytopenia >12 months (95% CI = 19.0–191.0, *p* < 0.01), having a first‐degree relative with thrombocytopenia (16 vs. 7, *p* < 0.01) and a higher platelet count nadir (67.9 ± 35.0 vs. 39.4 ± 33.9 × 10^9^/L, *p* < 0.05), were associated with genetic variants, suggesting these as indicators for genetic testing. This supports the R90's role in refining genetic testing criteria in thrombocytopenia.

## INTRODUCTION

Thrombocytopenia is found in a heterogeneous group of bleeding disorders with varying clinical implications.^1^, ^2^ In the paediatric population, a large proportion of patients presenting with isolated thrombocytopenia will be diagnosed with immune thrombocytopenia (ITP). ITP is an autoimmune disorder of peripheral platelet destruction. It is classically described as a diagnosis of exclusion; however, genetic testing is not routinely done at presentation. The role of genetic variants in inherited forms of thrombocytopenia has been established in recent years.^3^, ^4^ These genetic aberrations disrupt various pathways involved in platelet production, regulation and function. Consequently, it is crucial to consider genetic testing in the context of isolated thrombocytopenia in the paediatric population to establish an accurate diagnosis and appropriate treatment strategies.

The R90 bleeding and platelet disorders gene panel is a resource available to the NHS in England that allows for the assessment of potential genetic aberrations involved in non‐ITP (https://nhsgms‐panelapp.genomicsengland.co.uk/panels/545/v3.0). The panel also includes genes associated with platelet function and factor disorders. Specifically, the R90.1 panel consists of coagulation and platelet disorders WES or medium panel and the R90.2 consists of *F5*; *F11*; *MYH9*; *ENG*; *ACVRL1*; *F7*; *F8*; *F9*; *F10*; *VWF*, *MLPA* or equivalent.^5^ The current testing criteria include individuals with a bleeding or platelet disorder of likely monogenic aetiology where there are multiple possible causative genes.^5^ In our local practice, we suspect inherited thrombocytopenia disorders when patients exhibit persistent thrombocytopenia from early life, have a positive family history, show distinct characteristics typical of inherited conditions and/or fail to respond to first‐line treatments. It was also considered when a patient was not presenting with the typical history, examination and laboratory findings associated with ITP. Investigations that triggered consideration of a potential inherited platelet disorder were mean platelet volume (MPV) or diameter (MPD) when available or when a peripheral film identified an excessive abundance of large platelets. If there was no improvement in platelet counts, or if the response to initial treatments was suboptimal after 3–6 months, we reevaluated the likelihood of an inherited platelet disorder.

The integration of genetic testing resources in clinical practice comes with its set of challenges. The increased accessibility of such resources, like the R90 gene panel, has led to a surge in its clinical application. While this increased usage underscores the growing recognition of its value, it also necessitates a balanced approach to avoid the pitfalls of over‐testing. Appropriate and judicious use of genetic testing is important to optimise patient outcomes without incurring unnecessary investigations. This study aims to provide evidence to refine referral criteria for testing and to assess the current diagnostic utility of the R90 gene panel for those with a thrombocytopenia disorder.

## METHODOLOGY

A retrospective, single‐centre service evaluation of patients from Manchester Royal Infirmary (MRI) and Royal Manchester Children's Hospital (RMCH) who underwent the R90 bleeding and platelet disorders gene panel between October 2018 and June 2023. The inclusion criteria were patients who presented with thrombocytopenia, defined as platelet count <100 × 10^9^/L. Patients referred for the R90 panel with a suspected platelet function or factor disorder were excluded. Patient demographics, including bleeding history, ethnicity, age, family history and thrombocytopenia characteristics, such as length of thrombocytopenia and response to any previous ITP treatment were collected. Family history is defined as thrombocytopenia in a first‐degree relative. A response to treatment for ITP, including steroids, thrombopoietin receptor agonists and intravenous immunoglobulin (IVIg), was defined as platelet counts >30 × 10^9^/L and at least a doubling of the baseline count while a complete response was defined as a platelet count exceeding 100 × 10^9^/L. Participants consented to data use for service evaluations before genetic screening referral.

### Classification and description of genetic variants

Genetic variants identified in genes in the R90 panel are classified according to the American College of Medical Genetics and Genomics and the Association for Clinical Genomic Science (ACGS) best practice guidelines for variant interpretation.^6^, ^7^, ^8^, ^9^ Variants that are classed as pathogenic and likely pathogenic are reported on the genetic report using Human Genome Variation Society (HGVS) nomenclature. Genetic changes that are classed as variants of uncertain significance are included in the interpretive report if further evidence, such as segregation of the variant with phenotype within a family, or functional studies, may assist in promoting the classification to likely pathogenic. Genetic reports follow the ACGS best practice guidelines.^10^

### Statistical analysis

Statistical analysis was carried out for the variables investigated. Data were analysed using either an unpaired two‐tailed *t*‐test or a chi‐square, with two degrees of freedom. For both tests, the significance level was set at *p* < 0.05. The statistical analyses were performed using Prism Graph 9 (GraphPad Software, Boston, Massachusetts, USA).

## RESULTS

**FIGURE 1:**
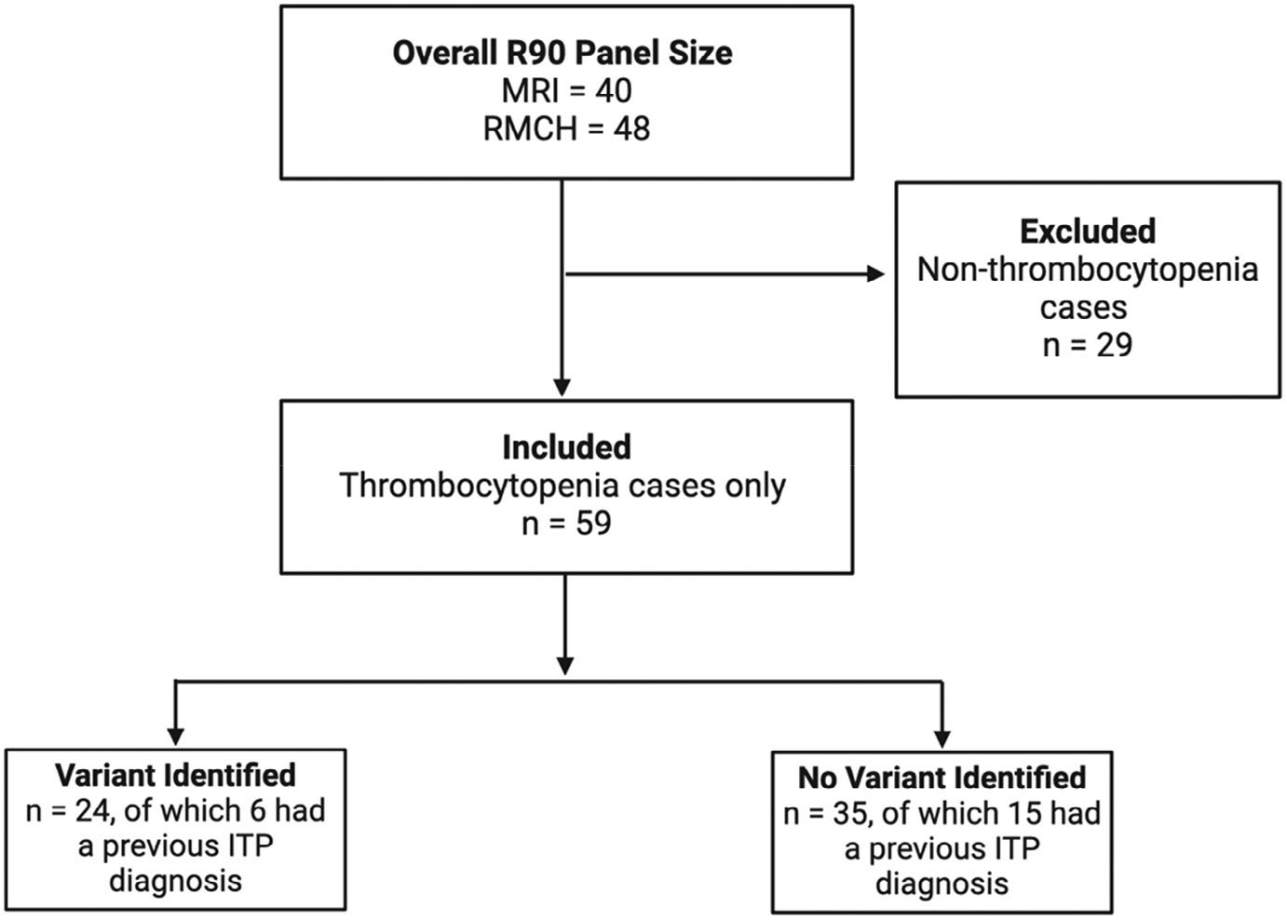
Flowchart of patient inclusion for thrombocytopenia cases only (*n* = 59). MRI, Manchester Royal Infirmary; RMCH, Royal Manchester Children's Hospital.

A total of 88 individuals were identified as undergoing the R90 panel, of which 41 (46.6%) had a genetic variant identified. Of the 88 individuals, 59 (67.0%) were referred due to suspected hereditary thrombocytopenia and were included in the following analysis (Figure 1; Data S1).

### Patient characteristics

A genetic variant was noted in 24 of the 59 (40.7%) referred thrombocytopenia cases. Of those, 43 of the 59 (72.9%) were females and the mean age was 21.5 years (range 0.4–69.0 years). The most common bleeding symptoms noted across all patients were bruising (52.5%), menorrhagia (27.1%), petechia (20.3%), epistaxis (11.9%) and bleeding after dental extraction/gum bleeding (8.5%) (Data S2).

ITP had been previously diagnosed in six (25.0%) patients in whom the R90 panel identified a genetic variant. Two cases within this group had a complete or partial response in platelet count after IVIg. In one case, a patient exhibited a genetic anomaly in the *ABCG8* gene linked to sitosterolaemia and platelet dysfunction.^11^ It is suspected that the patient had previously experienced ITP, which led to an acute drop in platelet count to 2 × 10^9^/L. This condition responded to IVIg, but the counts returned to a thrombocytopenic baseline. Another case involved a genetic defect in the *PRKACG* gene, associated with macrothrombocytopenia.^12^ This patient, who was also experiencing iron deficiency anaemia and COVID‐19 at the time of presentation, exhibited thrombocytopenia initially believed to have been ITP. For this patient, there were challenges in data verification due to the information being obtained from translated documentation. The patient later relocated overseas. Due to the inability to verify these data, this patient was excluded from all further analyses.

### Correlations with genetic testing outcomes

Thrombocytopenia greater than 12 months (95% CI = 19.0–191.0 months, *p* < 0.01) and family history of thrombocytopenia (16 of 24 vs. 7 of 35, *p* < 0.01) were associated with a higher probability of a genetic variant being identified. The mean platelet count nadir was notably higher in cases where a variant was identified (64.1 ± 26.0 × 10^9^/L) than in those where there was none (38.5 ± 34.0 × 10^9^/L), demonstrating a statistically significant difference (*p* < 0.01). There was a statistically significant association between platelet counts >15 × 10^9^/L and a higher likelihood of a genetic variant being identified (*p* < 0.05).

**FIGURE 2:**
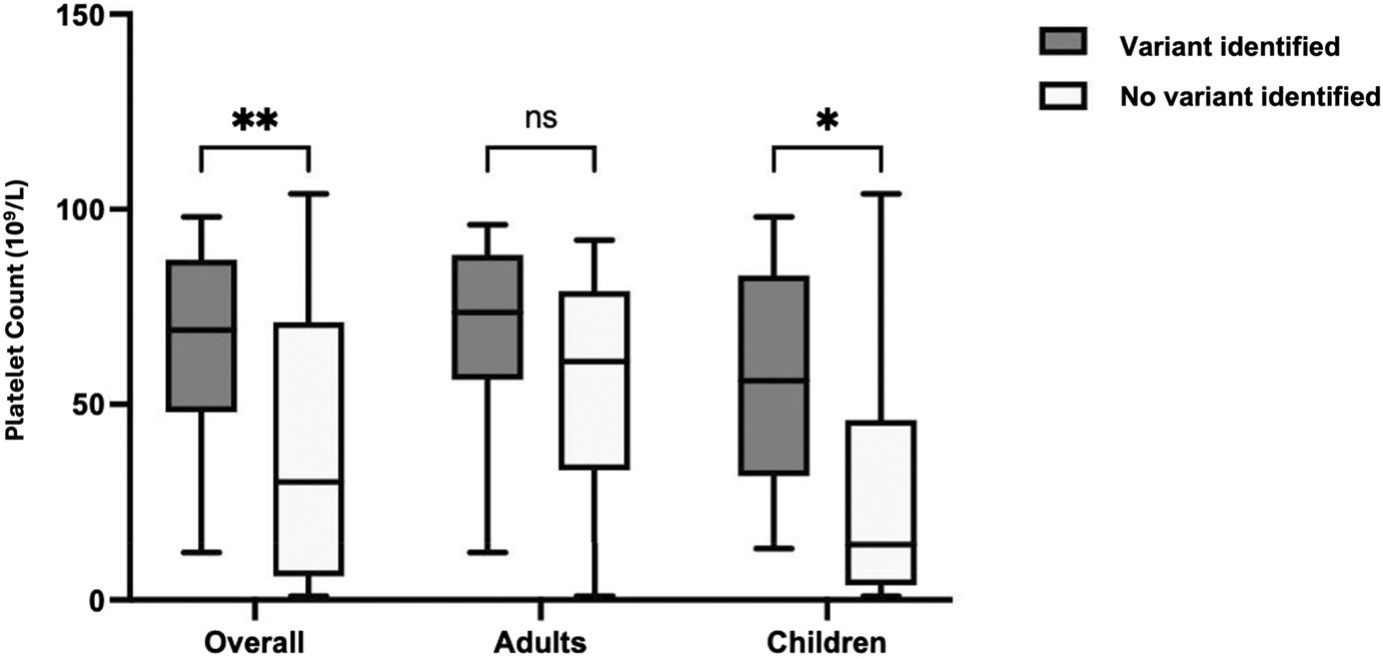
Platelet count nadir and genetic test result of overall, adult and children cases. *p < 0.05, **p < 0.01, ns = not significant.

Further analysis by age groups revealed that adults had significantly higher mean platelet counts (61.8 ± 26.1 × 10^9^/L) compared to children (37.2 ± 35.0 × 10^9^/L), with a *p*‐value of less than 0.01 (Figure 2). Among children, those with no variant identified exhibited significantly lower platelet count nadir (28.9 ± 34.4 × 10^9^/L) than those with a variant (57.7 ± 29.0 × 10^9^/L, *p* < 0.05). Conversely, in the adult group, the difference in mean platelet count nadir between those without (54.8 ± 27.3 × 10^9^/L) and those with a variant (68.2 ± 24.1 × 10^9^/L) was not statistically significant (*p* = 0.19).

Associations between the identification of a variant and gender, ethnicity and previous response to ITP treatment did not reach significance. This may have been due to the limited power because of the small sample size.

## DISCUSSION

The overall finding of a variant in 46.6% (41/88) of cases tested for the R90 panel from this service evaluation is higher than that reported by a similar study at 33.5%.^13^ This study involved both adults and children with bleeding disorders of unspecified cause and non‐ITP. The findings underscore the R90 panel's effectiveness in identifying genetic variants responsible for these conditions, further supporting its diagnostic utility.

Of the 59 individuals referred solely due to platelet count‐related screening in RMCH and MRI, 40.7% (24/59) had a variant identified. This aligns with another study's reported rate of 47.8%.^14^ This study utilised high‐throughput sequencing to analyse patients with diverse bleeding, thrombotic and platelet disorders. It revealed that nearly half of the patients diagnosed with platelet count disorders exhibited detectable gene variants, highlighting the role of genetic testing in accurately diagnosing and managing these conditions. This observation can serve as a potential indicator to clinicians about which patient cohort may benefit most from the R90 panel, specifically when focusing on platelet count‐related screening.

It was noted that ITP had been previously diagnosed in 25.0% of those with a variant identified. This observation suggests the possibility that some cases initially labelled as ITP are congenital thrombocytopenia. It is important not to miss potential thrombocytopenia syndromes, especially those associated with malignancy or the development of bone marrow failure. Additional genetic screenings, such as the R91 cytopenia (https://nhsgms‐panelapp.genomicsengland.co.uk/panels/519/v3.0) and the R15 primary immunodeficiency (https://nhsgms‐panelapp.genomicsengland.co.uk/panels/519/v3.0) gene panels, may be beneficial in distinguishing these cases, offering a more targeted diagnostic and therapeutic approach.

Key findings from this study that were associated with the detection of a variant were duration of thrombocytopenia greater than 12 months, family history of thrombocytopenia and platelet count nadir. This was particularly significant in the paediatric population where children with a higher platelet count nadir were more likely to have a variant identified. These findings align with the observation that lower platelet count nadirs are typically associated with ITP rather than genetic causes, with ITP often characterised by mean platelet count nadir around 15 × 10^9^/L.^15^ Our analysis suggests that patients with a nadir platelet count of less than 15 × 10^9^/L are significantly less likely to have a genetic variant identified on R90 testing. If validated, it would be beneficial for clinicians to consider a referral for genetic testing in patients with the above characteristics. This criterion can serve as a potential marker to increase the likelihood of identifying a genetic cause, which could significantly impact patient management and diagnostic precision (Data S3).

Gender, ethnicity and previous response to ITP treatments were not associated with the finding of a genetic variant. The results of this study do not support using these as referral criteria for R90 panel testing for patients with thrombocytopenia.

## CONCLUSION

In routine clinical practice, the R90 gene panel has a clinically important yield of genetic variants. It has been proven to be valuable for identifying genetic causes of thrombocytopenia. Prolonged thrombocytopenia duration, the presence of family history and a higher platelet count nadir may be considered potential indicators for genetic evaluation.

## AUTHOR CONTRIBUTIONS

GG and LR collected and analysed the data. GG, LR, MS, JT and JG wrote the main manuscript text. All authors reviewed the manuscript.

## FUNDING INFORMATION

The authors declare that no funding was received for this manuscript.

## CONFLICT OF INTEREST STATEMENT

The authors declare that they have no conflicts of interest.

## ETHICS APPROVAL STATEMENT

Ethics approval was not required for this study as patient consent was obtained for the use of their data for service evaluation as part of their referral for genetic testing.

## Supporting information


Data S1.

Data S2.

Data S3.

## Data Availability

All data supporting the findings of this study are available within the paper and its Supplementary Information.
