# Supplementary material for: Service evaluation of R90 bleeding and platelet disorders gene panel in thrombocytopenia cases
Source: Br J Haematol. 2024 Dec 9;206(3):930–4. doi: 10.1111/bjh.19947 (PMC11886932; doi:10.1111/bjh.19947)
Supplement: Supplementary file 1 — Data S1. [file BJH-206-930-s003.docx]

**Supplemental Data 1. Characteristics Of Patients Referred for R90 Gene Panel**

| **Gene** | **Genetic Classification** | **Bleeding History** | | **Patient demographics** | | | | | **Disease Characteristics** | | **Previous ITP Treatment** | | |  |
| --- | --- | --- | --- | --- | --- | --- | --- | --- | --- | --- | --- | --- | --- | --- |
|  |  |  |  | **Age** | | **Gender** | **Ethnicity** | **Family History of Thrombocytopenia** | **Lowest platelet count (10^9^/L)** | **Duration of thrombocytopenia (months)** | **Previous Diagnosis of ITP** | **Treatment** | **Response** |  |
| **POSITIVE CASES** | | | | | | | | | | | | | |  |
| *ABCG8* | Pathogenic | Bruising, Epistaxis | | 28 | | Male | White British | Yes | 2 | 132 | Yes | Steroids | NR |  |
|  |  |  |  |  |  |  |  |  |  |  |  | IVIg | R |  |
| *GATA1* | Pathogenic | None | | 69 | | Female | White British | Yes | 64 | 72 | No |  |  |  |
| *ANKRD26* | Likely pathogenic | None | | 43 | | Female | White British | Yes | 92 | 288 | No |  |  |  |
| *GFI1B* | Likely pathogenic | Bleeding after extraction | | 22 | | Male | Mixed White and Asian | Yes | 87 | 8 | No |  |  |  |
| *HPS1* | Pathogenic | Epistaxis, Menorrhagia | | 53 | | Female | White British | Yes | 75 | 360 | No |  |  |  |
| *GP1BB* | Variant of Unknown Significance | Menorrhagia | | 34 | | Female | Asian Chinese | Yes | 96 | 120 | No |  |  |  |
| *ACTN1* | Likely pathogenic | Menorrhagia | | 46 | | Female | White British | Yes | 48 | 288 | Yes | Steroids | NR |  |
|  |  |  |  |  |  |  |  |  |  |  |  | IVIg | NR |  |
| *MYH9* | Likely pathogenic | Menorrhagia | | 43 | | Female | Other Asian Background | Yes | 72 | 420 | No |  |  |  |
| *ACTN1* | Variant of Unknown Significance | Bleeding after extraction, Menorrhagia | | 46 | | Female | White Irish | Yes | 63 | 144 | No |  |  |  |
| *ACTN2* | Variant of Unknown Significance | Not stated | | 58 | | Female | White British | Yes | 12 | 120 | No |  |  |  |
| *GP1BA* | Pathogenic | Bruising | | 43 | | Male | White European | Yes | 94 | 492 | No |  |  |  |
| *ABCG8* | Pathogenic | Bruising, Menorrhagia | | 24 | | Female | Prefer not to say | Yes | 80 | 6 | Yes | No |  |  |
| *MPL* | Pathogenic | Bruising, Menorrhagia, Petechial rash | | 16 | | Female | White British | No | 33 | 12 | Yes | No |  |  |
| *MECOM* | Variant of Unknown Significance | None | | 18 | | Male | White British | Yes | 59 | 7 | No |  |  |  |
| *WAS* | Pathogenic | Bleeding | | 2 | | Male | White British | No | 56 | 12 | No |  |  |  |
| *PRKACG* | Variant of Unknown Significance | Bruising, Petechial rash | | 6 | | Female | Asian Pakistani | Yes | 13 | 5 | Yes | IVIg | R |  |
| *DNAJC21* | Pathogenic | None | | 5 | | Female | Asian Pakistani | No | 33 | 12 | Yes | IVIg | NR |  |
| *MYH9* | Pathogenic | Bruising, Gum bleeding | | 10 | | Female | White British | Yes | 96 | 84 | No |  |  |  |
| *RUNX1* | Pathogenic | Bruising | | 7 | | Female | White British | No | 70 | 9 | No |  |  |  |
| *MYH9* | Likely pathogenic | None | | 8 | | Female | Asian Chinese | Yes | 68 | 96 | No |  |  |  |
| *GP1BB* | Variant of Unknown Significance | Bruising, Gum bleeding, Menorrhagia | | 27 | | Female | Asian Pakistani | Yes | 75 | 12 | No |  |  |  |
| *ITGA2B* | Pathogenic | Bruising | | 8 | | Female | White British | Probable |  | 84 | No |  |  |  |
| *GP1BA* | Likely pathogenic | Bruising, Petechial rash | | 14 | | Female | Asian Pakistani | No | 30 | 84 | No |  |  |  |
| *ACTN1* | Variant of Unknown Significance | Bruising | | 13 | | Female | White British | No | 98 | 120 | No |  |  |  |
| **NEGATIVE CASES** | | | | | | | | | | | | | | |
|  | |  | Bruising, Epistaxis | 28 | | Female | | White British | No | 42 | 60 | Yes | Steroids | NR |
|  | |  | Not stated | 29 | | Female | | White British | No | 69 | 12 | No |  |  |
|  | |  | None | 17 | | Female | | Asian Pakistani | Yes | 104 | 24 | No |  |  |
|  | |  | Not stated | 18 | | Female | | White European | No | 22 | 3 | Yes | Steroids | NR |
|  |  |  |  |  |  |  |  |  |  |  |  |  | IVIg | NR |
|  | |  | Bruising, Epistaxis, Menorrhagia | 22 | | Female | | White British | Yes | 77 | 96 | No |  |  |
|  | |  | Menorrhagia | 19 | | Female | | White British | No | 30 | 108 | No |  |  |
|  | |  | Menorrhagia | 54 | | Female | | White British | No | 36 | 372 | No |  |  |
|  | |  | Bruising | 40 | | Female | | White European | No | 71 | 252 | No |  |  |
|  | |  | Bruising, Menorrhagia | 41 | | Female | | Asian Indian | Yes | 81 | 12 | No |  |  |
|  | |  | Bruising, Menorrhagia | 44 | | Female | | White British | Yes | 61 | 10 | No |  |  |
|  | |  | Bruising, Petechial rash | 9 | | Male | | White British | No | 15 | 48 | Yes | No |  |
|  | |  | Bruising | 14 | | Female | | Asian Bangladeshi | No | 38 | 36 | No |  |  |
|  | |  | None | 10 | | Female | | Prefer Not To Say | No | 70 | 24 | No |  |  |
|  | |  | Bruising, Menorrhagia, Petechial rash | 14 | | Female | | Prefer Not To Say | No | 20 | 7 | Yes | No |  |
|  | |  | None | 45 | | Female | | Other Ethnic Group | No | 92 | 60 | No |  |  |
|  | |  | Petechial Rash | 13 | | Female | | White British | No | 2 | 12 | Yes | Eltrombopag | CR |
|  | |  | Bruising | 5 | | Male | | Other Ethnic Group | No | 9 | 24 | Yes | Steroids | NR |
|  | |  | Bruising, Epistaxis, Menorrhagia | 13 | | Female | | White British | No | 1 | 24 | Yes | Romiplostim | CR |
|  | |  | None | 2 | | Female | | Asian Pakistani | No | 6 | 7 | No | Steroids | R |
|  | |  | Bruising | 9 | | Female | | White British | No | 7 | 96 | Yes | Romiplostim | CR |
|  | |  | Bruising, Epistaxis, Petechial Rash | 12 | | Male | | White British | Yes | 1 | 12 | Yes | Romiplostim | CR |
|  | |  | Bruising, Epistaxis, Petechial Rash | 16 | | Female | | White British | No | 1 | 12 | Yes | No |  |
|  | |  | Petechial Rash | 14 | | Female | | White British | No | 13 | 96 | Yes |  |  |
|  | |  | None | 15 | | Male | | White British | No | 99 | 2 | No | Steroids | NR |
|  | |  | Bleeding after extraction | 2 | | Male | | Other Asian Background | No | 2 | 4 | Yes | No |  |
|  | |  | Bruising, Petechial Rash | 1 | | Male | | White European | No | 34 | 9 | No |  |  |
|  | |  | Bruising, Menorrhagia | 15 | | Female | | Asian Pakistani | No | 25 | 16 | No | No |  |
|  | |  | Bruising | 2 | | Male | | Asian Indian | No | 3 | 3 | Yes | No |  |
|  | |  | Bruising, Petechial Rash | 7 | | Male | | White British | No | 4 | 4 | Yes |  |  |
|  | |  | Bruising, Petechial Rash | 15 | | Female | | White British | No | 5 | 1 | No |  |  |
|  | |  | Bruising | 16 | | Male | | Prefer Not To Say | No | 82 | 6 | No |  |  |
|  | |  | None | 6 | | Male | | Asian Pakistani | No | 90 | 60 | No |  |  |
|  | |  | Bruising | 53 | | Female | | White British | No | 49 | 216 | No | No |  |
|  | |  | Bruising | 4 | | Male | | White British | No | 71 | 60 | Yes |  |  |
|  | |  | None | 0.4 | | Female | | Prefer Not To Say | No | 16 | 4 | No |  |  |
