# Supplementary material for: Service evaluation of R90 bleeding and platelet disorders gene panel in thrombocytopenia cases
Source: Br J Haematol. 2024 Dec 9;206(3):930–4. doi: 10.1111/bjh.19947 (PMC11886932; doi:10.1111/bjh.19947)
Supplement: Supplementary file 2 — Data S2. [file BJH-206-930-s002.docx]

**Supplemental Data 2. Characteristics Of Patients with Thrombocytopenia**

| **Patient Characteristics** | **Size**  **(n = 59)** |
| --- | --- |
| Genetic Test |  |
| *Variant identified* | 24 (40.7) |
| *No variant identified* | 35 (59.3) |
| Gender |  |
| *Male* | 16 (27.1) |
| *Female* | 43 (72.9) |
| Family History of Thrombocytopenia |  |
| *Yes* | 22 (37.3) |
| *No* | 37 (62.7) |
| Ethnicity |  |
| *White British and Europeans* | 36 (61.0) |
| *Non-white British and Europeans, or not specified* | 23 (38.9) |
| Bleeding History |  |
| *Bruising* | 31 (52.5) |
| *Menorrhagia* | 16 (27.1) |
| *Petechia* | 12 (20.3) |
| *Epistaxis* | 7 (11.9) |
| *Bleeding after extraction/ gum bleeding* | 5 (8.5) |
