# Supplementary material for: Service evaluation of R90 bleeding and platelet disorders gene panel in thrombocytopenia cases
Source: Br J Haematol. 2024 Dec 9;206(3):930–4. doi: 10.1111/bjh.19947 (PMC11886932; doi:10.1111/bjh.19947)
Supplement: Supplementary file 3 — Data S3. [file BJH-206-930-s001.docx]

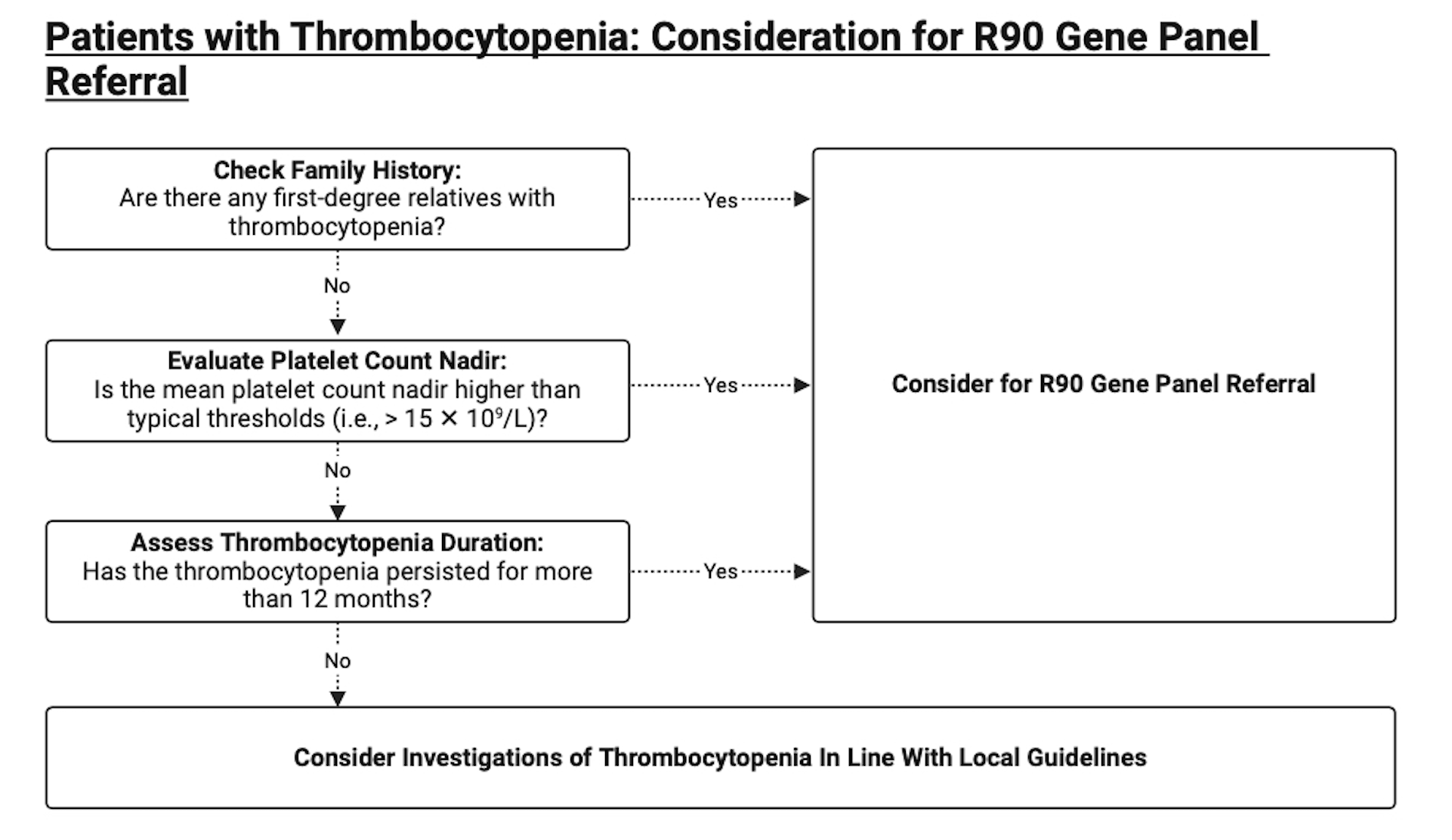
**Supplemental Data 3. Algorithm for Consideration for R90 Gene Panel Referral for Patients with Thrombocytopenia**
